# Artificial intelligence assisted simulation and surgical video analytics for ophthalmic surgery training and competence development

**DOI:** 10.3389/fmed.2026.1781818

**Published:** 2026-03-19

**Authors:** Minghui Zhao, Juan Li, Shuang Li, Jiali Liu, Yanyun Jiang, Xiaoling Lai, Juan Yang, Lan Pang, Lilan Tang, Kunke Li, Ligang Jiang

**Affiliations:** 1 Department of Ophthalmology, Shanghai Municipal Hospital of Traditional Chinese Medicine, Shanghai University of Traditional Chinese Medicine, Shanghai, China; 2 Department of Ophthalmology, Fuyong People's Hospital, Shenzhen, China; 3 Shenzhen Eye Hospital, Shenzhen Eye Medical Center, Southern Medical University, Shenzhen, China; 4 Department of Ophthalmology, Quzhou People’s Hospital, Quzhou Affiliated Hospital of Wenzhou Medical University, Quzhou, Zhejiang, China

**Keywords:** artificial intelligence, clinical competence development, Dreyfus model of skill acquisition, ophthalmic surgery training, surgical video analytics

## Abstract

Based on the Dreyfus model of skill acquisition, this article classifies the professional development of ophthalmologists into four stages: novice, advanced beginner, competent, and expert. In this review, artificial intelligence (AI) is operationally defined as data-driven algorithms that enable prediction, perception, and objective assessment from multimodal surgical data. We distinguish AI methods from immersive hardware, such as virtual reality (VR), which serves as a training interface that may or may not incorporate AI-driven assessment and feedback. Accordingly, this manuscript focuses on AI-enabled simulation, computer-vision-based surgical video understanding, and registry/EHR-driven clinical practice and training continuum. At the novice stage, AI-enabled assessment within VR simulation helps trainees form muscle memory and standardized operating habits. This is achieved through haptic-enabled modules and objective performance metrics. For advanced beginners, computer-vision models and attention-visualization techniques support surgical workflow understanding and structured debriefing, assisting trainees in building surgical logic and spatial cognition. When doctors reach the competent stage, AI uses large-scale clinical data to estimate complication risk and support scenario-based crisis training, strengthening complication management and non-technical skills. At the expert stage, AI-assisted surgical video analytics can benchmark technique patterns and surface potential blind spots, facilitating continuous calibration and knowledge sharing. Overall, the evidence to date suggests that AI is best positioned as an assistive tool to augment human learning and decision-making. However, generalizability, interpretability, data governance, and medicolegal accountability remain key barriers to safe and scalable deployment.

## 1 Introduction

“The entire art of medicine lies in observation”, and “guiding a person onto the right path is the only thing you can do” (1). For a long time, the training of ophthalmologists has followed the classic “apprenticeship” model established by William Halsted, with the core principle of “seeing one, doing one, and teaching one” (2). However, in recent years, microsurgery has become increasingly sophisticated. Patients’ safety awareness has risen steadily. Medical data has grown explosively. Together, these changes pose unprecedented challenges to the traditional training model. That model relies on accidental case exposure to accumulate experience and uses subjective judgment to pass on skills. It is no longer sufficient for the demands of modern ophthalmic surgery (3).

Ophthalmic surgery, especially cataract and retinal surgery, requires surgeons to perform precise operations at the micrometer level in extremely narrow anatomical spaces (4, 5). For beginners, such high intensity operational requirements often exceed their cognitive load threshold instantly. When working memory is completely occupied by instrument manipulation and anatomical structure recognition, learners have no energy left for in depth strategic thinking (6).

This article focuses on the cognitive maturity of doctors. Drawing on the classic Dreyfus model of skill acquisition (7), it classifies the professional development of ophthalmologists into four distinct competence levels: Novice, Advanced Beginner, Competent, and Expert. While the original Dreyfus model delineates five stages, including a proficient stage between competent and expert, this review adopts a four stage framework tailored to the specific context of ophthalmic surgical training. In microsurgical practice, the transition from proficiency to expertise often presents a continuum rather than a discrete leap. Furthermore, current artificial intelligence (AI) applications in surgery tend to target either foundational skill acquisition or high level global analysis, making a combined discussion of the advanced stages more practical for this technological review (8, 9). It is crucial to clarify that these “stages” refer to qualitative levels of proficiency rather than fixed chronological phases (7). In this hierarchical framework, competence development is strictly stage dependent; a trainee does not advance automatically with time, but only transitions to the next stage when specific cognitive and operational thresholds are met. If a surgeon fails to demonstrate the required proficiency, such as moving from rule based execution to situational context perception, they will be considered to remain in the current stage, no matter how many years they have practiced. Within this framework, this review explores how doctors can build different AI technology integration and application systems tailored to these specific competence levels, and delves into how technology can provide accurate assistance to doctors at every key node of their cognitive development (Table 1).

**Table 1:** Stage based framework: how AI supports ophthalmic surgical competence development (Dreyfus model).

Dreyfus stage	Dominant training bottleneck (in surgery training)	AI roles in training	Typical AI methods/Systems mentioned in the manuscript	Primary training outputs	Clinical validation status	Level of evidence	Key limitations
Novice	High cognitive load; difficulty building stable hand eye coordination and safe “muscle memory”; hard to parse expert workflows into discrete steps	(1) AI-enabled objective scoring and adaptive difficulty adjustment within haptic-feedback VR simulators for low-risk deliberate practice; (2) AI-driven semantic decomposition of surgical workflow using computer vision; (3) Automated performance feedback based on AI motion tracking and structured rubrics to prevent early formation of bad habits	VR surgical simulators with force/haptic feedback (e.g., Eyesi); AI enabled haptic simulation; computer vision phase/step recognition and semantic segmentation; video based objective skill assessment aligned with structured rubrics (e.g., OSCAR)	Standardized instrument handling; reduced unnecessary motion; reproducible step execution; early error prevention	Established Standard (Mandatory in many global residency curricula)	Level I	(1) High capital cost and maintenance fees. (2) Limited haptic realism (tactile feedback) compared to wet labs. (3) Restricted anatomical variations.
Advanced beginner	Sees steps but struggles to infer surgical logic, attention allocation, and spatial/anatomic relationships under the microscope	(1) AI-driven visual attention guidance to support pattern recognition and situational awareness development; (2) AI-based automated anatomical segmentation and 3D reconstruction to cultivate spatial cognition and functional anatomy understanding; (3) Structured AI-assisted post-hoc video debriefing to help trainees link surgical steps, goals, and risk cues, thereby strengthening intraoperative decision-making logic	Saliency/heatmap style visual guidance; multimodal visualization; AI-driven 3D anatomical segmentation & reconstruction	Faster acquisition of “where to look/what matters”; improved spatial understanding; earlier development of situational awareness; earlier development of pattern recognition ability and intraoperative decision-making logic	Validated (High accuracy in datasets; limited routine clinical deployment)	Level III-IV	(1) Black box nature: lacks explanatory feedback. (2) Performance degrades on poor-quality videos. (3) Data privacy issues with video storage.
Competent	Managing uncertainty: anticipating complications, intraoperative risk control, crisis handling; teamwork and non-technical skills under stress	(1) Risk prediction using registry/EHR/video data; (2) Early warning/navigation concepts; (3) Crisis simulation and structured debriefing with objective logs	Big data predictive analytics (e.g., registry based signals); AR/navigation concepts; multi-user VR simulation; objective logs for debriefing	Better complication anticipation and management; improved decision making under stress; improved communication/leadership	Experimental/Prototype (Mostly research settings; few commercial approvals for full AI guidance)	Level III-IV	(1) Processing latency can disrupt surgical flow. (2) Registration errors due to rapid eye movements. (3) Potential cognitive distraction.
Expert	Skill drift and blind spots hidden by intuition; need for continuous calibration, benchmarking, and dissemination	(1) Fine grained surgical video analytics to surface blind spots; (2) Benchmarking outcomes and technique; (3) Crowdsourced datasets/platforms for global sharing	Video based quantitative analytics; crowdsourced surgical video datasets (e.g., CATARACTS Challenge); registry based outcome benchmarking	Continuous skill calibration; quality improvement; knowledge dissemination and standard setting	Widely Adopted (Integrated into standard biometry devices; Standard of Care)	Level II	(1) Dependent on high-quality input data (biometry). (2) Proprietary algorithms prevent external auditing. (3) Less accurate for rare pathologies.

Level of evidence: categorized based on the Oxford Centre for Evidence-Based Medicine (OCEBM) criteria. Level I: randomized controlled trials (RCTs); Level II: retrospective cohort or comparative studies; Level III: case–control or diagnostic accuracy studies; Level IV: Case series or feasibility studies.

This review is intentionally centered on AI methods that support ophthalmic surgical training and perioperative decision support through simulation based assessment, computer-vision analysis of surgical videos, and data driven risk prediction using registry/EHR information. Although outside the main scope of this review, broader ophthalmic education research has also reported promising applications of virtual simulation in non-surgical teaching contexts (10), suggesting a wider role for immersive digital learning environments across ophthalmology. While robotic microsurgery, autonomous control, and fully integrated intraoperative navigation are important directions, a systematic treatment of these domains is beyond the scope of the present manuscript and is highlighted as future work (Table 2).

**Table 2:** AI applications in ophthalmic surgery training and practice, organized by surgical phase.

Surgical phase	Training focus	AI application	Representative methods	Validation status
Preoperative/simulation	Motor skill acquisition	AI-scored VR simulation	Haptic modeling, performance metrics	Widely validated
Intraoperative (learning)	Workflow understanding	Phase recognition, saliency maps	CNN, attention visualization	Validated, limited deployment
Intraoperative (decision)	Risk control	Early warning systems	Registry-driven ML models	Experimental / prototype
Postoperative	Outcome benchmarking	Video analytics, registry comparison	Motion analysis, cohort statistics	Retrospective validation
Longitudinal	Continuous improvement	Blind spot detection	Video-based analytics	Emerging

## 2 Conceptual framework and definitions

**Figure 1:**
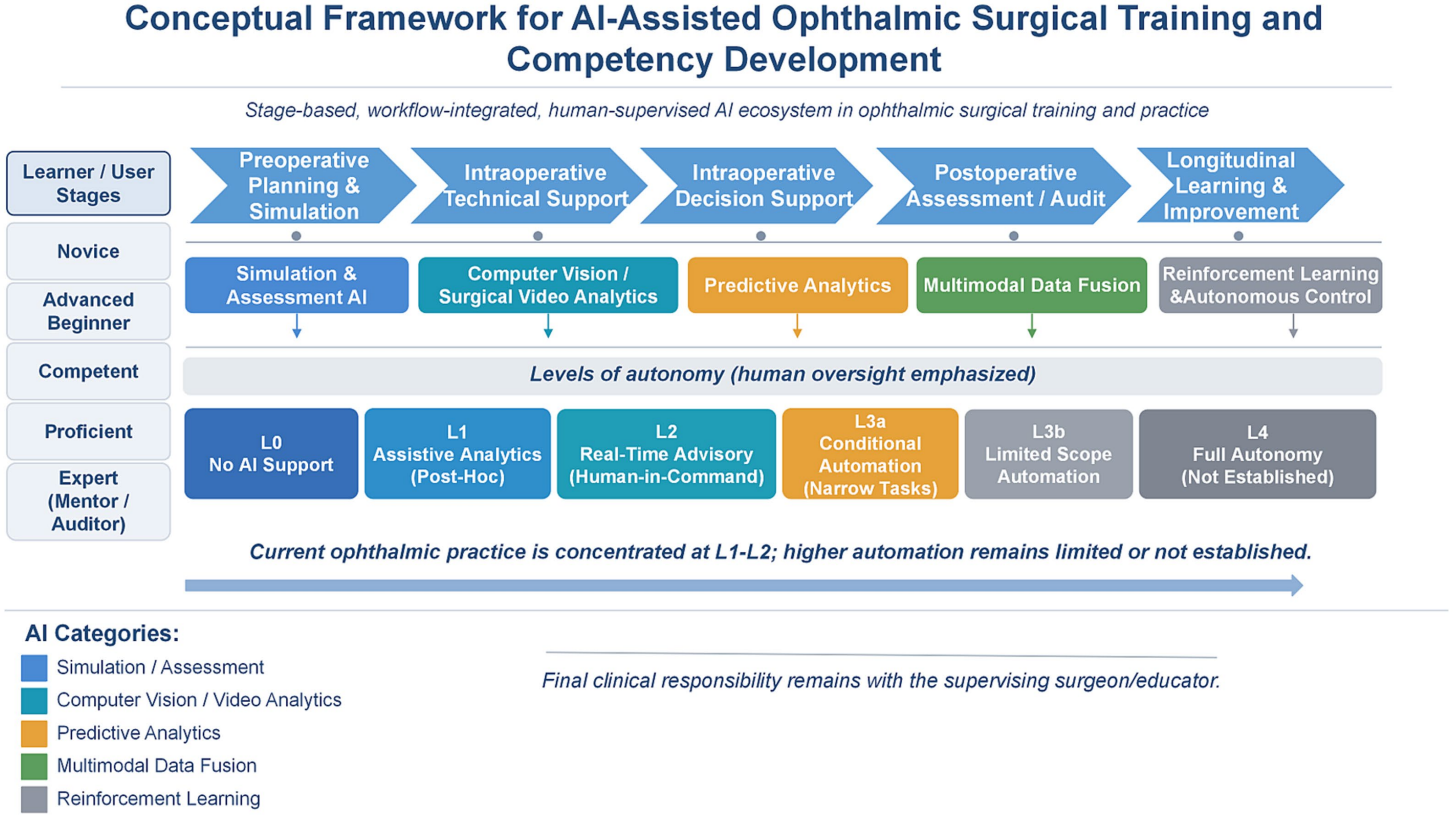
Conceptual framework of AI-assisted ophthalmic surgical training and competence development. This conceptual framework illustrates a stage-based, workflow-integrated, and human-supervised AI ecosystem for ophthalmic surgical training and practice, covering preoperative planning/simulation, intraoperative technical and decision support, postoperative assessment/audit, and longitudinal learning/improvement. It integrates learner/user stages, representative AI methodological categories, and autonomy levels (L0–L4) with emphasis on human oversight. L3a denotes conditional automation for narrow tasks, L3b denotes limited-scope automation, and L4 (full autonomy) is not established in current ophthalmic surgical training/practice. The framework highlights that current practice is concentrated at L1–L2 and that final clinical responsibility remains with the supervising surgeon/educator. Colors in the AI-category row denote methodological categories, whereas colors in the autonomy row indicate conceptual progression rather than validation status.

In this manuscript, AI refers to data-driven computational methods, including machine learning and deep learning, that learn patterns from data to perform prediction, perception, and/or objective assessment. In ophthalmic surgery training and practice, we focus on three practical AI roles: (i) simulation/assessment intelligence, including algorithmic scoring, feedback generation, and proficiency tracking within simulators; (ii) computer-vision intelligence, such as phase recognition, tool/action segmentation, and post-hoc explanation visualization from surgical video; (iii) predictive analytics, for example, risk estimation, or outcomes modeling using registry/EHR data. We explicitly distinguish AI algorithms from enabling interfaces such as virtual reality (VR) and augmented reality (AR). VR or AR are delivery platforms that may incorporate AI, but are not themselves AI. In the context of ophthalmic surgery training and competence development, this review focuses on supervised deep learning-based AI paradigms. Other AI paradigms such as reinforcement learning and embodied AI are not discussed due to their limited clinical application in this field to date (Figure 1).

## 3 Methodology

To insure a comprehensive review, a literature search was conducted on PubMed, Web of Science, and IEEE Xplore databases for articles published between January 2014 and December 2025. Keywords included “artificial intelligence,” “ophthalmic surgery training,” “surgical simulation,” “deep learning,” and “Dreyfus model.” We included original research, systematic reviews, and conference proceedings focusing on AI applications in ophthalmology resident training and skill assessment. Articles solely focused on diagnostic AI without surgical relevance were excluded. A total of 86 relevant studies were selected for synthesis (see Table 3).

**Table 3:** Categorization of AI methodologies in ophthalmic surgical training and their validation status.

AI methodology	Primary use	Evidence level	Clinical readiness	Key limitations
Supervised deep learning (CV)	Skill assessment	Level II–III	Moderate	Domain shift
Predictive analytics (registry/EHR)	Risk estimation	Level II	Limited	Interpretability
Multimodal fusion	Cognitive load estimation	Level III–IV	Experimental	Data integration
Reinforcement learning	Skill optimization	Level IV	Conceptual	Safety, ethics

## 4 Novice stage: motion capture and tactile reshaping

### 4.1 Tactile simulation training to help form instinctive bodily responses for surgical operations

Traditional Wet Lab training uses animal eyeballs as the operating medium. This novice stage primarily maps to preoperative education and simulation-based skill acquisition, where repeated low-risk practice and structured feedback prepare trainees before entering the operating room. This approach is not only costly, but also fails to provide standardized operational feedback (11). In contrast, virtual reality (VR) surgical simulators provide structured, repeatable practice in a controlled environment, and can generate standardized performance metrics. On their own, VR systems deliver the immersive interface and physics-based haptic rendering. The intelligence layer comes from AI-enabled assessment modules embedded within these simulators. These modules perform functions such as automated motion tracking, objective performance scoring against expert benchmarks, real-time proficiency classification, and adaptive difficulty adjustment based on the trainee’s evolving skill profile. When coupled with AI-enabled assessment modules, these platforms can further deliver objective, granular feedback to support targeted deliberate practice (12, 13). A study by Liu et al. (14) confirmed that repeated training on such simulators can help surgeons effectively transfer the operational skills they have learned to real surgical scenarios.

VR hardware provides the interface and haptic feedback based on physics engines. AI algorithms serve a distinct and complementary role. They objectively score trainee performance by comparing instrument trajectories, force profiles, and completion times against expert benchmarks. They also adaptively adjust training difficulty based on real-time proficiency analysis (15, 16). Taking capsulorhexis as an example, this is a core operation in cataract surgery. When a novice operates in a virtual training environment, the VR system renders the visual scene and generates baseline haptic resistance through its physics engine. AI algorithms then analyze multimodal signals, including instrument kinematics and tissue deformation patterns. Based on this analysis, AI modules tune simulation parameters, escalate task complexity for proficient trainees, and generate structured performance reports that highlight specific areas for improvement. This multi-modal input combining vision and touch helps novices establish a mapping relationship between the two in their brains. In this way, the AI component transforms a passive simulation exercise into a guided, adaptive training session, thereby efficiently consolidating muscle memory in low-risk virtual scenarios (17, 18). For instance, Thomsen et al. showed that novices trained on the Eyesi simulator achieved a 50% reduction in posterior capsule rupture rates during their first real world surgeries compared to traditionally trained residents (19). Similarly, systematic reviews indicate that VR training reduces the time to complete specific tasks by approximately 20–30% in subsequent wet-lab evaluations (20). However, the magnitude of benefit may vary by curriculum design, baseline trainee experience, and the specific complication endpoint.

However, it is critical to note that even validated systems struggle to replicate the complex tissue instrument interactions of pathological eyes, such as zonular dialysis. Consequently, they remain an adjunct to, rather than a replacement for, traditional wet-lab training where haptic fidelity is paramount.

High-fidelity simulators are now a standard for cataract training, offering validated modules for capsulorhexis. In contrast, vitreoretinal modules, while available, face greater challenges in replicating the nuanced tissue-instrument interactions required for membrane peeling, though recent advancements are bridging this gap.

### 4.2 Computer vision for semantic decomposition of surgical actions

Another core difficulty for novices is their inability to break down the coherent and smooth surgical operations of experts into standardized step by step actions (21). To address this issue, AI driven computer vision technology can realize semantic segmentation of surgical videos. With the algorithm support of Convolutional Neural Networks (CNN), AI systems can automatically analyze a complete cataract surgery video and accurately decompose it into a series of independent operation steps, such as incision making, capsulotomy, and water separation (22, 23).

**Figure 2:**
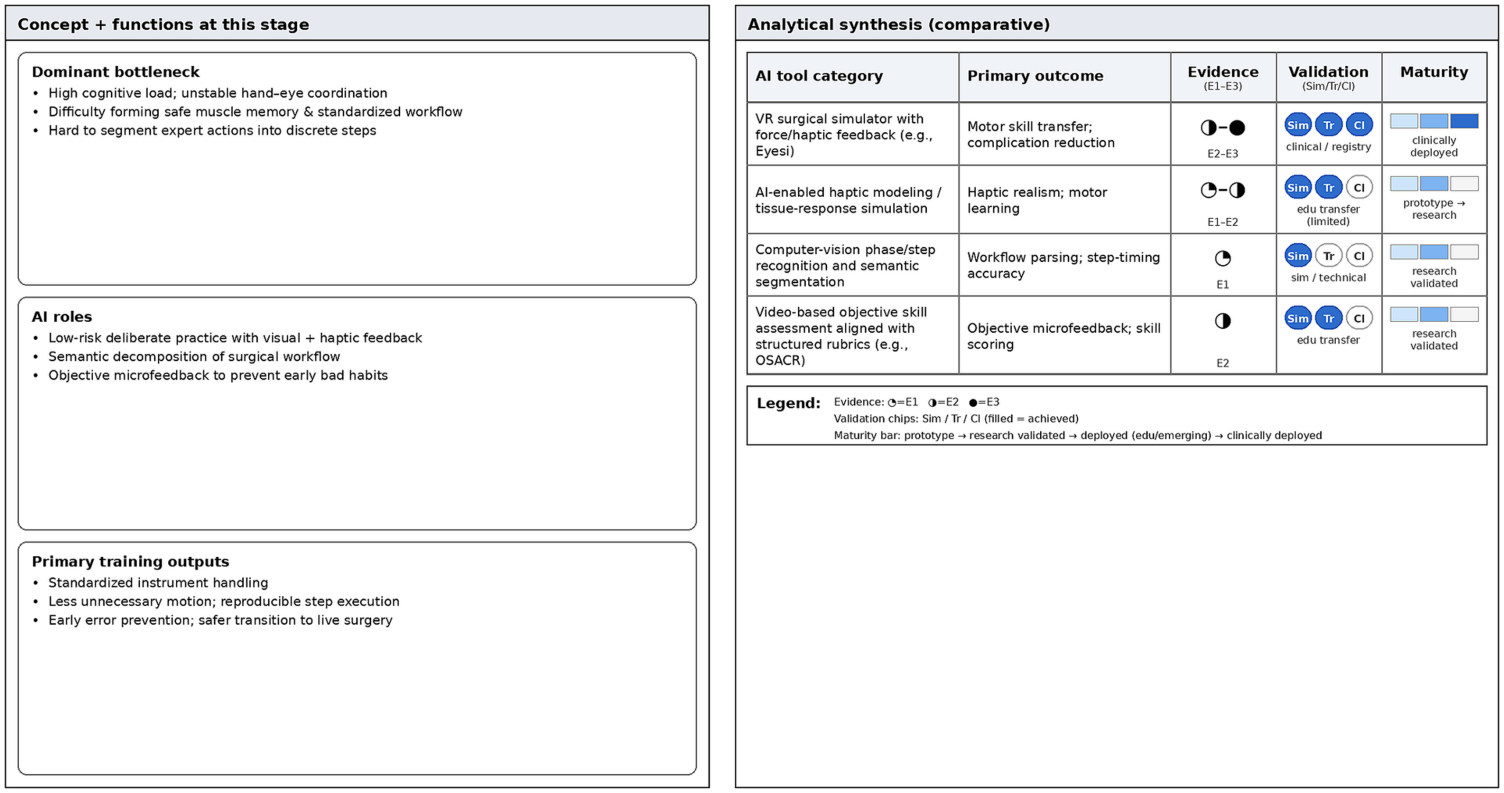
Novice stage—evidence dashboard. Comparative summary of AI tools for early motor-skill acquisition and workflow formation. The dashboard synthesizes primary outcome domains, evidence tier (E1–E3), validation status, and maturity level across tool categories.

Based on the above mentioned method of decomposing surgical actions, AI systems can conduct quantitative evaluations of surgical operations at the micro level. For instance, the evaluation model developed by Kim et al. (24) is supported by temporal convolutional network technology. It can accurately score the key operation step of capsulotomy by referring to the ophthalmic surgical competency assessment rubric (OSCAR) evaluation criteria established by the International Council of Ophthalmology (ICO) (24). This model can conduct millisecond-level detailed analysis on the smoothness of surgical actions and the rationality of instrument operation paths. This real time and objective feedback mechanism can effectively prevent novices from forming bad surgical operation habits in the early stage of training. It functions as a virtual guidance mentor available around the clock (24) (Figure 2).

However, current CNN-based approaches face inherent limitations. They require massive, high quality annotated datasets which are labor intensive to produce. Furthermore, these models often struggle with domain shift, which means that they may perform well on training data from one medical center but fail when lighting conditions or instrument types change in another hospital (21). Unlike traditional apprenticeship where mentors provide context aware feedback, current AI algorithms may flag technical deviations without understanding the surgeon’s adaptive intent in complex cases.

Traditionally, mentorship relies on subjective observation. The attending surgeon watches a procedure and provides verbal feedback based on personal experience. This approach is limited by time constraints and inter-observer variability. In contrast, AI-based computer vision analysis provides objective scoring. It quantifies surgical actions at the millisecond level against standardized rubrics. This offers a scalable complement to human supervision, particularly in high-volume training programs where one-on-one mentoring time is scarce (25).

## 5 Advanced beginner stage: from seeing operations to insight into surgical logic

This advanced beginner stage maps most directly to intraoperative workflow cognition and post-case debriefing, where trainees learn to recognize surgical phases, key cues, and error patterns from operative videos and guided review. When doctors complete the accumulation of basic surgical operation skills and enter the advanced beginner stage, the focus of their cognitive training gradually shifts from simple hand manipulation of instruments to the development of surgical operation logic and intraoperative decision making thinking. Advanced beginners need to learn to establish meaningful connections between scattered operation steps, surgical goals, and anatomical structure risks (26). Unlike the Novice stage, where AI primarily supports motor skill formation through haptic simulation and action decomposition, the advanced beginner stage presents a different cognitive challenge. Trainees at this level can execute individual steps but struggle to perceive the logic connecting those steps. They need to develop three core abilities: (1) pattern recognition of critical surgical structures, (2) situational awareness in dynamic operative fields, and (3) the capacity to allocate visual attention based on surgical priorities rather than random scanning. For advanced beginners, AI tools specifically target this cognitive leap from seeing to perceiving. This allows the trainee to reallocate mental resources toward establishing the logic of surgical flow and recognizing situational patterns. These are the defining criteria for transitioning out of the beginner stage. For example, they need to understand the matching relationship between a specific incision angle and the subsequent lens extraction path, or the correlation logic between instrument movement speed and retinal protection.

Unlike the Novice stage, for advanced beginners, AI tools specifically target the cognitive leap from seeing to perceiving. This allows the trainee to reallocate mental resources toward establishing the logic of surgical flow and recognizing situational patterns, which are the defining criteria for transitioning out of the beginner stage.

At this stage, the functional positioning of AI also changes. In the Novice stage, AI acts as an action simulation and assessment tool. Here, it becomes a tool for disassembling surgical operation logic and guiding cognitive connections. It trains doctors to not only see surgical steps with their eyes, but also gain insight into the surgical principles and risk control logic behind the operations with their brains.

### 5.1 Directional guidance of visual attention in surgical visualization

The first core ability that advanced beginners must develop is learning where to look during surgery. This is a prerequisite for pattern recognition and situational awareness. In the training of ophthalmic surgical operations related to fundus structures, such as vitrectomy for retinal detachment or macular hole repair, the value of AI is not only reflected in assisting with preoperative lesion localization. It also lies in providing real-time visual guidance for key surgical steps during the operation, directly training the trainee’s visual attention allocation. With the help of Saliency Maps technology in the field of deep learning, which is a visualization technique that highlights the pixels in an image most relevant to the model’s decision-making process, AI can integrate intraoperative optical coherence tomography (OCT) images and surgical microscopic views, then highlight critical surgical targets and risk areas on the real-time operation interface (27, 28). During retinal membrane peeling surgery, AI can mark the boundary between the epiretinal membrane and normal retinal tissue, or highlight the location of fragile retinal blood vessels that are prone to bleeding during manipulation (29). This targeted visual highlighting helps advanced beginners quickly focus on the core areas that require precise operation, avoiding the problem of being distracted by complex anatomical structures and missing key operation points.

For advanced beginners, this real-time surgical visual guidance has high teaching value. It directly supports the three core competencies required at this stage. First, it trains pattern recognition. By repeatedly highlighting the boundary between pathological and normal tissue, AI helps trainees internalize what critical structures look like under varying surgical conditions. Second, it cultivates situational awareness. The trainee learns to identify key operation targets and potential risks from the dynamic surgical field, rather than passively viewing the entire operative area (30). Third, it lays the groundwork for intraoperative decision-making. When a trainee can reliably perceive where the risks are, they begin to anticipate which actions are safe and which require caution. This cognitive progression, from guided perception to independent judgment, is precisely the developmental milestone that defines the advanced beginner stage. AI does not replace the surgeon’s judgment of surgical steps. Instead, it accelerates the formation of a surgical visual cognitive model. By enhancing the visual prominence of key structures, AI helps trainees align their visual focus with the operational requirements of complex fundus surgeries more quickly. Over time, this guided attention becomes internalized. The trainee no longer needs the AI overlay to know where to look. At that point, the advanced beginner is ready to transition toward competent-level practice, where the challenge shifts from “what to see” to “what to decide” (31, 32).

Although deep learning models have great potential in research environments, real-time saliency map overlays are currently limited by processing delays and registration errors caused by rapid eye movements. Therefore, most of the current applications are still in the emerging phase, mainly used for post-hoc video analysis to debrief trainees, rather than as live intraoperative guidance systems.

It is worth noting that the maturity of these technologies varies by subspecialty. In anterior segment surgery, for example, cataract, phase recognition is relatively robust due to the structured nature of the procedure (33). In contrast, vitreoretinal surgery involves greater variability in tissue deformation and illumination, making AI-based instrument tracking and guidance more experimental and less reliable in real-world clinical deployment compared to traditional supervision (3).

### 5.2 Anatomy reconstruction and spatial thinking cultivation in virtual environments

In addition to the ability to recognize two dimensional planar images, advanced beginners also need to establish a three dimensional (3D) spatial pathological cognitive model (34). Traditionally, this relies on mental reconstruction, but AI-driven automated segmentation and reconstruction technologies are transforming this learning process. In fields like neuro-ophthalmology, AI algorithms, specifically deep learning models based on U-Net architectures, can automatically extract complex anatomical structures from raw MRI or CT data. These algorithms rapidly segment the optic nerve, extraocular muscles, and surrounding vasculature, generating high-fidelity 3D meshes that serve as the foundation for virtual environments (35).

**Figure 3:**
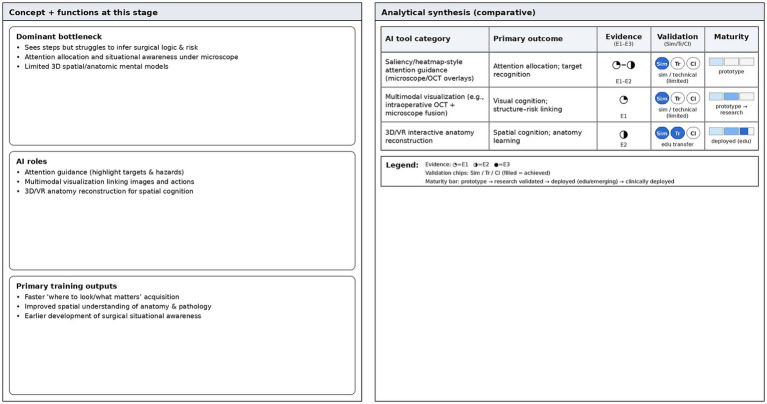
Advanced beginner stage—evidence dashboard. Comparative summary of AI tools supporting attention guidance, situational awareness, and spatial/anatomic cognition. The dashboard summarizes outcome domains, evidence tier (E1–E3), validation status, and maturity level.

By integrating these AI-generated models into immersive systems, doctors can virtually enter the interior of the eyeball. They can observe the spatial relationships between the optic nerve and surrounding blood vessels or simulate tumor compression pathways with patient specific precision (36). Unlike generic anatomical maps, these AI-reconstructed models connect abstract theoretical knowledge with specific clinical phenotypes. This process helps advanced beginners transition from mechanical memorization of anatomical positions to an in-depth understanding of functional anatomy, powered by the algorithm’s ability to visualize invisible spatial logic (Figure 3).

## 6 Competent stage: risk prediction and complications management

When doctors enter the competent stage, they are already proficient in performing routine surgeries. This stage aligns with perioperative decision-making across preoperative planning and postoperative risk surveillance, where the focus shifts from executing steps to anticipating complications and responding to non-routine scenarios. At this point, the core cognitive challenge is no longer the execution of a single action, but the management of uncertainty and decision making in complex situations. At this stage, the role of AI has undergone a qualitative change, evolving from a simple instructor to a real-time collaborator. It uses big data and predictive analysis to improve doctors’ ability to predict risks (37, 38).

At this pivotal transition, the trainee moves beyond executing standardized steps to managing uncertainty. Unlike the advanced beginner stage, where AI guides what to see for the competent surgeon, AI shifts to supporting what to decide. By providing real-time probabilistic risk assessments, AI acts as a cognitive guardrail, allowing the surgeon to develop the emotional stability and hierarchical decision-making skills necessary for independent practice.

### 6.1 Predictive analysis and intraoperative crisis early warning

In complex ophthalmic surgeries, even experienced doctors find it difficult to remain alert to all potential risk factors at all times. Big data analytics and machine learning algorithms applied to registries can identify weak signals before complications occur. These weak signals include subtle instrument tremors that indicate fatigue, or minor fluctuations in anterior chamber stability. A study based on the IRIS (Intelligent Research in Sight) registry database shows that AI can analyze data from millions of cataract surgeries and identify specific risk patterns related to rare complications such as intraocular inflammation (36, 39).

While fully autonomous systems remain experimental, emerging research suggests that future operating rooms may integrate AI-supported tools with augmented reality (AR) to serve as intraoperative navigation assistants (40, 41). Conceptually, such systems could monitor the distance between instruments and the retina, or assess the risk of corneal endothelial damage in real time. For instance, in complex scenarios like hard lens nuclei or relaxed suspensory ligaments, prototype algorithms are being designed to issue warnings before dangerous maneuvers occur, just like analogous to a car’s collision alert. Although clinical validation is still needed, this type of support aims not to deprive doctors of their decision-making power, but to potentially help them make more rational judgments in high-pressure environments by providing additional dimensions of information (42, 43).

Nevertheless, these technologies are largely conceptual or experimental in clinical practice. The black box nature of these algorithms raises unresolved liability issues. If an AI fails to predict a complication, the legal responsibility remains undefined. Furthermore, false-positive alerts during surgery could dangerously distract a competent surgeon, a phenomenon known as automation bias.

### 6.2 Stress testing and nontechnical skills cultivation in simulated environments

Another important training content in the competent stage is dealing with unexpected situations. Traditional training rarely provides doctors with opportunities to practice repeatedly how to handle emergencies such as severe bleeding or vitreous prolapse. However, modern VR simulators can create customized crisis scenarios in virtual environments. By introducing multi-user VR collaborative training, the lead surgeon can conduct team training exercises with anesthesiologists and nurses in virtual space (44).

**Figure 4:**
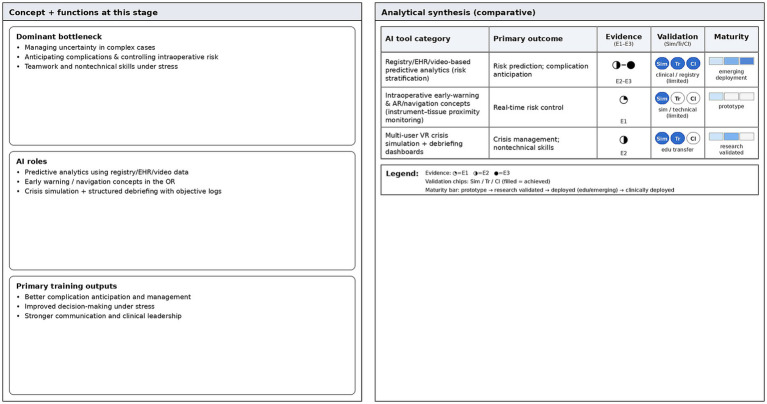
Competent stage—evidence dashboard. Comparative summary of AI tools for risk prediction, complication anticipation, and crisis/nontechnical skills training. The dashboard contrasts evidence tier (E1–E3), validation status, and maturity level to highlight validation gaps.

The value of this kind of training goes beyond the simple technical operation level and extends to the cultivation of non-technical skills. The key among these skills is the shaping of situational awareness and the improvement of closed loop communication capabilities (45, 46). The AI system can record data such as the team’s emergency response time, information communication frequency, and decision execution sequence in the background. After the drill is completed, it generates multi-dimensional review data. This kind of feedback based on objective data plays a key role in cultivating doctors’ psychological resilience and clinical leadership in extremely high pressure environments (Figure 4).

## 7 Expert stage: self reflection and algorithmic mirroring

Even doctors who have reached the expert level still face challenges in maintaining their skills, preventing skill degradation, and adapting to new technologies. This stage maps to continuous quality improvement across the full surgical workflow (pre-, intra-, and postoperative), where expert performance is refined through benchmarking, audit-and-feedback, and knowledge transfer using longitudinal clinical and video data. At this stage, the role of AI is an objective mirror and calibrator. It helps experts break through ability bottlenecks or maintain their peak state through data driven self reflection (47).

Through granular motion analytics, AI reveals sub-perceptual inefficiencies that even a human mentor might miss. This feedback loop is essential for breaking through the plateau of competence to achieve the fluidity and minimal cognitive effort distinctive of the expert stage.

### 7.1 Surgical video analysis and blind spot detection

Experts often rely on intuition to operate. This is both an advantage and a hidden risk, because intuition can sometimes cover up subtle technical deviations. AI driven surgical video analysis technology can mine and present key information and in-depth insights that are difficult for human observers to perceive. By conducting in-depth learning on a large number of surgical videos, AI can conduct quantitative analysis on every action of experts, such as the movement distance of instruments in the eye, the frequency of ineffective actions, and even the amplitude of subtle tremors (48).

Preliminary studies indicate that this kind of analysis has the potential to reveal blind spots or habitual biases that experts may not be aware of themselves. For example, AI models might detect that an expert shows slightly lower cognitive efficiency when dealing with tissues in a specific quadrant. This could manifest as increased hesitation time or redundant instrument movements. AI might also detect a decrease in operational stability when the surgeon is fatigued. If validated by further research, this kind of objective feedback could enable senior doctors to make fine adjustments to their techniques, achieving a high-level form of deliberate practice.

### 7.2 Crowdsourced knowledge and global experience sharing

Experts are not only learners, but also creators and disseminators of knowledge (49). AI platforms promote the crowdsourcing and sharing of global ophthalmic experience. Through public dataset projects such as the CATARACTS Challenge, surgical videos from experts around the world are collected, annotated, and analyzed (21). With this collective wisdom, an expert can learn from the experience of peers on the other side of the world without face to face communication.

**Figure 5:**
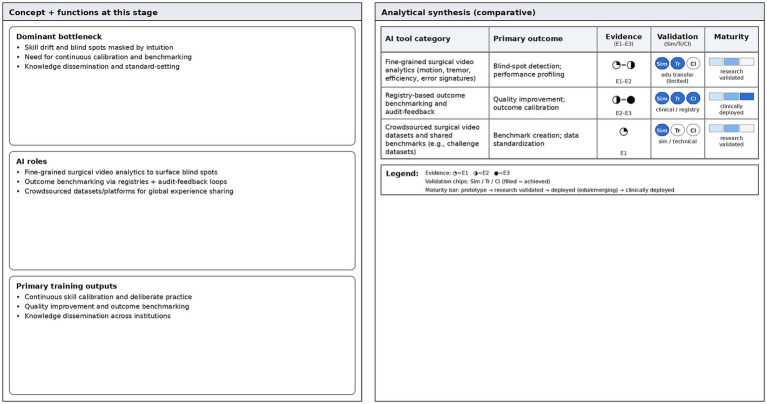
Expert stage—evidence dashboard. Comparative summary of AI tools for performance profiling, continuous calibration, and outcome benchmarking (e.g., video analytics and registry-based audit-feedback). The dashboard compares evidence tier (E1–E3), validation status, and maturity level. Evidence tier is encoded using circular markers: E1 = technical feasibility/algorithm validation; E2 = learner performance or skill-transfer outcomes; E3 = clinical or registry-level outcomes. Validation status is indicated by tier markers (simulation-only, educational transfer, clinical/registry). Maturity level is represented by a three-level bar (prototype, research-validated, clinically deployed), with color intensity increasing with deployment level.

In addition, big data based registration system allow experts to compare their surgical results with those of global peers (50). This kind of comparison is not for competition, but to provide a broader reference system. It helps experts position their clinical performance in a larger scope and ensures that they always stay at the forefront of the industry (Figure 5).

## 8 Cost–benefit analysis

From a cost-effectiveness perspective, the initial investment for high-fidelity simulators is substantial, with systems like the VRmagic Eyesi costing significantly more than basic wet-lab setups. However, cost-utility analyses suggest that preventing a single major complication, such as a dropped nucleus, can offset the cost of training multiple residents, making it economically viable for large institutions over the long term (51).

## 9 Challenges and ethical considerations

Although the prospects are bright, the integration of AI is not without risks. Algorithmic bias is a significant concern. If AI models are trained predominantly on data from specific ethnic groups or device manufacturers, they may fail to generalize to broader populations (52). Furthermore, there is a risk of skill degradation if trainees become over-reliant on AI guidance. Legally, the liability for surgical errors guided by AI suggestions remains an unresolved grey area. Institutional Review Boards and professional societies must establish clear guidelines to balance innovation with patient safety.

## 10 Conclusion and future directions

Artificial intelligence is progressively reshaping ophthalmic surgical training from experience-dependent apprenticeship toward objective, data-driven competence development. Across the novice to expert continuum, current evidence supports the role of AI-enabled simulation, computer-visio, and registry-driven predictive modeling as assistive tools that enhance feedback consistency, accelerate skill acquisition, and enable scalable benchmarking. Importantly, these systems function most effectively when aligned with clearly defined educational objectives and human oversight, rather than as autonomous decision-makers.

Looking forward, the next phase of AI evolution in ophthalmic surgery will extend beyond task-specific models toward integrated, multimodal intelligence frameworks that operate across the full surgical lifecycle.

### 10.1 Multimodal foundation models for surgical understanding

Future AI systems are expected to transition from single-modality video analysis to multimodal foundation models that jointly learn from surgical video, instrument kinematics, force-feedback signals, intraoperative OCT, and perioperative clinical data. For training, this may enable richer competency assessment that integrates technical precision, temporal efficiency, and cognitive load. However, robust external validation and standardized data interfaces will be essential before these models can be safely deployed at scale.

### 10.2 Digital twins of ocular anatomy and personalized simulation

Another promising direction is the development of digital twins of ocular anatomy, constructed from patient-specific imaging and continuously updated surgical data. Such virtual replicas could allow surgeons to rehearse procedures under realistic biomechanical conditions, explore alternative strategies, and anticipate complication pathways before entering the operating room. For trainees, digital twins may bridge the gap between generic simulation and individualized anatomy, accelerating the transition from rule-based execution to situational reasoning.

### 10.3 Reinforcement learning for surgical strategy optimization

Rather than imitating expert behavior alone, reinforcement learning based systems could explore action and outcome trade-offs in simulated environments to identify safer or more efficient procedural pathways. In the near term, such approaches are most appropriately confined to offline simulation and training scenario design, where safety risks are minimal. However, translation into real-world assistance will require stringent safeguards, transparent reward structures, and clear boundaries that preserve surgeon authority.

### 10.4 Federated learning and cross-center collaboration

Sustainable progress in AI-assisted surgical education will depend on access to diverse, high-quality data. Federated learning provides a viable pathway to train robust models across institutions while preserving patient privacy and data sovereignty. For ophthalmic surgery, federated infrastructures could reduce center-specific bias, improve generalizability across devices and populations, and facilitate international benchmarking of training outcomes. More broadly, recent predictive modeling studies in other ophthalmic subspecialties have likewise highlighted the importance of heterogeneous datasets and external validation, reinforcing the need for cross-center collaboration before AI models can be adopted as dependable decision-support tools (53).

Beyond the scope of this review, AI-integrated robotic assistance and closed-loop control for ophthalmic microsurgery represent an important parallel track that will require dedicated evidence synthesis and prospective clinical validation.

In summary, AI-assisted ophthalmic surgical training is entering a transition from isolated tools toward interconnected intelligence systems. The greatest impact will likely arise not from full automation, but from thoughtfully designed human–AI collaboration that augments perception, supports reflection, and reinforces clinical responsibility. Future research should prioritize prospective, multi-center studies that link AI-enabled training interventions to long-term patient outcomes, thereby ensuring that technological advancement translates into meaningful clinical benefit.
